# Unmet social needs of community-living older adults with dementia: A scoping review

**DOI:** 10.1177/13872877261442966

**Published:** 2026-04-23

**Authors:** Sunny Tan, Marine Markaryan, Óscar Ribeiro, Rita Maldonado Branco, Liliana Sousa

**Affiliations:** 1 Department of Education and Psychology, RISE-Health, 56062 University of Aveiro, Aveiro, Portugal; 2 Department of Psychiatry and Neuropsychology, Mental Health and Neuroscience Research Institute, Alzheimer Center Limburg, 5211 Maastricht University, Maastricht, Netherlands; 3 Department of Communication and Art, Research Institute for Design Media and Culture (ID+), Health+Design Lab (HEAD), 56062 University of Aveiro, Aveiro, Portugal

**Keywords:** Alzheimer's disease, community-living, dementia, interpersonal relations, needs assessment, older adults, social participation

## Abstract

Dementia-related cognitive decline and ageing-related challenges impact social participation and relationships. Limited knowledge of dementia among family, friends and the public hinder support for interactions with those affected. Unmet social needs in community-living older adults with dementia remain underrecognized, despite their importance in healthy ageing and quality of life. This scoping review mapped existing evidence on unmet social needs of community-living older adults with dementia, including how these needs vary across dementia stages. We conducted a scoping review following Preferred Reporting Items for Systematic Reviews and Meta-Analyses extension for Scoping Reviews (PRISMA-ScR) and Joanna Briggs Institute (JBI) guidelines. Four databases were searched (2000 –April 2025) in English, Portuguese and Mandarin, yielding 24 included articles. Seven themes of unmet social needs were identified: (i) participation in out-of-home activities, (ii) support for maintaining independence in daily life (iii) companionship, (iv) meaningful relationships, (v) respectful and dignified social interactions, (vi) emotional support, and (vii) social needs during the pandemic. Unmet social needs differed by dementia stage: in mild dementia, unmet social needs mostly relate to out-of-home activities, while in moderate and severe stages unmet social needs shifted towards daily activities, companionship and emotional support. Stigma emerged as a transversal influence shaping experiences of unmet social need across stages and contexts. Unmet social needs evolve across dementia stages and are influenced by relational, socio-cultural and structural factors. Future research should include more advanced stages, diverse populations, and strategies for addressing social needs from their own perspectives.

## Introduction

The World Health Organization's (WHO) Decade of Healthy Ageing defines “healthy aging” as “the process of developing and maintaining the functional ability that enables well-being in older age.”^1^ Functional abilities encompass the capacity to meet one's basic needs; learn, grow, and make decisions; maintain mobility; contribute to society; and build and maintain relationships. The ability to engage in social activities and maintain interpersonal connections is therefore a fundamental component of healthy aging.

Drawing on the WHO decade of Healthy Ageing framework,^1^ we conceptualize social needs as opportunities for social participation and engagement, including the ability to maintain meaningful interpersonal relationships and social activities. In addition, the Social Production Functions theory recognizes three core social needs^2,3^: *affection, behavioral confirmation and status*. Affection is defined as the need to give and receive love independent of personal abilities or resources. Behavioral confirmation refers to the need to feel that one's actions are ‘‘right’’ by oneself and others, and to have a sense of belonging with groups with shared values. Status relates to the need to distinguish oneself from others based on individual abilities, roles or resources.^2^

Based on these frameworks, *unmet social needs* are operationalized as: i) constraints that limit engagement in social activities; ii) hinderances to the maintenance of interpersonal relationships; and/or iii) barriers to the fulfilment of the core social needs.

Dementia, a neurocognitive disorder affecting more than 55 million people worldwide, poses several challenges to social interaction and activities participation not only for people with dementia and their caregivers, but also for friends, wider social networks, and others within the care constellation.^4,5^ Dementia is characterized by a decline in cognitive functioning, with impairments in areas such as memory, language, and social cognition.^6^ Behavioral and psychological symptoms of dementia (BPSD) like apathy, agitation and anxiety can further hinder social engagement.^7,8^ Literature suggests that older adults with dementia tend to report more unmet social needs than those without cognitive impairment.^9^ During the mild stages of dementia, individuals can take part in community or social activities without help and may appear unimpaired to those who do not know them well.^10^ In the moderate stage, signs and symptoms progress and become increasingly restricting.^10^ In the severe stage, affected individuals may have complete dependence, inactivity, serious memory disturbances and physical signs. Consequently, maintaining social connections becomes increasingly difficult throughout the disease trajectory. In addition to cognitive decline, other aging-related factors further reduce social interaction. Retirement often leads to decreased daily contact with colleagues, while physical frailty and age-related health issues contribute to reduced mobility.^11^ The loss of relatives, spouses, and close friends can further exacerbate social isolation, limiting opportunities to build new relationships.^11,12^

Studies focusing on community-living older adults with dementia highlight numerous unmet intrapersonal and interpersonal social needs, particularly related to daytime activities (e.g., social, work, leisure, learning activities), receiving company (e.g., social contact, contact with friends), and receiving help with psychological distress (e.g., agitation, needs for sympathy and support).^13–16^ Recognizing and addressing the unmet social needs of community-living older adults with dementia is crucial, as they are associated with a lower health-related quality of life and more symptoms of depression.^13,14,17^

Two main measures have been used to assess unmet needs among older adults with dementia. The Camberwell Assessment of Needs for the Elderly (CANE) is one of the most commonly used tools for identifying met and unmet needs among older adults. It comprises 24 areas related to the care recipient and two areas focused on the caregiver,^16,18^ covering areas such as social contact, accommodation, physical health, and access to information about their health condition. The CANE is typically administered by healthcare professionals and may not fully capture the subjective experiences of those living with dementia. Another widely used tool is the John Hopkins Dementia Care Needs Assessment (JHDCNA), which comprises seven areas related to the care recipient and six areas focused on the caregiver. The JHDCNA captures unmet social needs such as social engagement and interpersonal relationships within the Daily Activities domain, and it also addresses other areas including health care financing and general health care needs. Developed by clinical dementia care experts, the JHDCNA is clinician-administered, which may also limit the subjective inputs of those living with dementia.^14^ Although qualitative methods such as interviews are also used to explore unmet needs among older adults with dementia, the majority of research in this area remains quantitative.^12^

Previous reviews have mapped the evidence on the broader spectrum of unmet needs among older adults with dementia. A scoping review by Morrisby et al.^19^ and a meta-analysis by Curnow et al.,^20^ identified needs in areas such as information, self-care, and peer support. However, none of the reviews^19,20^ focused specifically on unmet social needs. Furthermore, a large part of the existing literature is based on the perspectives of caregivers or healthcare professionals, rather than on older adults with dementia themselves.^9,20–22^ Although proxy reporting may be required in later stages of dementia due to reduced insight or capacity to complete questionnaires, dependence on proxy reports alone may fail to capture subjective social needs that are best understood from the perspective of older adults living with dementia.^21^ Additionally, the current literature also does not provide clear insights on whether variations exist in unmet social needs across the stages of dementia.

To address these gaps, we conducted a scoping review to map the existing evidence and identify gaps in the literature about the unmet social needs of community-living older adults with dementia, considering their own perspectives. Our primary research question was: (1) “What are the unmet social needs of community-living older adults with dementia considering their perspective?”. The secondary research question was: (2) “How those unmet needs evolve along the stages of dementia?”

## Methods

This review was conducted following the Joanna Briggs Institute (JBI) guidance for scoping reviews^23^ and is reported by the Preferred Reporting Items for Systematic reviews and Meta-Analyses extension for Scoping Reviews (PRISMA-ScR).^24^ Scoping reviews are a well-established method to identify key characteristics or factors of a given concept and research gaps.^25,26^ The protocol of this review was registered in the International Platform of Registered Systematic Review and Meta-analysis Protocols (https://inplasy.com/inplasy-2023-12-0082/).

The Population, Concept, Context (PCC) approach was used to outline inclusion and exclusion criteria (Table 1). Inclusion criteria consisted of primary peer-reviewed articles written in English, Portuguese or Chinese published from year 2000 onwards. The decision to focus on literature published in the past 25 years was to ensure relevance to today's context of care practices, social structures and cultural understandings of dementia, and align with the WHO's (2020) paradigm of healthy aging.^1^ It is acknowledged that some unmet social needs identified in studies conducted during this period may reflect changes in social interactions, expectations, and care arrangements, which differed significantly before, during and after the COVID 19 pandemic. A prior literature review on the subjective needs of people with dementia^27^ confirmed that most studies in which people with dementia were interviewed were conducted from year 2000 or later. All types of research approaches were considered. Unpublished studies and grey literature (e.g., reports and conference abstracts, books, documents) as well as systematic reviews or meta-analyses were excluded to ensure the scoping review provides an overview of available evidence based on peer-reviewed research.^26^

**Table 1.** Eligibility criteria according to the PCC approach.

	Inclusion criteria	Exclusion criteria
Population	Older adults (≥65 years old) diagnosed with dementia.	Other age groups Only caregivers
Concept	Unmet social needs, i.e., social and leisure activities, daytime activities, community life activities, engagement in meaningful occupation, social companionship, and interpersonal relationships.	Other needs Needs of caregivers only
Context	Community-living	Live in nursing care and residential homes, inpatient settings, or hospitals

### Search strategy

A search strategy was devised with the assistance of an academic librarian. An initial search on PubMed was conducted to explore and identify relevant English-language search terms aligned with the research questions for use in the search strategy. The same derived search terms were applied in all database searches, using the same filters and date ranges. Search terms included “older people” OR elderly OR “old person” OR “older adults”, dementia OR Alzheimer OR “Neurocognitive disorder”, “social needs” OR leisure OR occupation OR interact* OR activit*, home OR community OR “community-living”. Four online databases (PubMed, Scopus, Web of Science, and Academic Search Complete) were initially searched on 11 December 2023. The scoping review was updated with a final search conducted on 22 April 2025, across all previously used databases, with the exception of Academic Search Complete, which was no longer accessible through the institutional database subscriptions at the time of the search. Hand-search of key authors and citations of included studies was conducted to ensure all relevant studies were included. The search was supplemented by conducting manual citation searching of relevant papers, which resulted in 1 paper^16^ being added to the included studies. The full search strategy is provided in Supplemental Table 1.

### Screening procedure

Following the JBI recommendations, the screening was performed in sequential steps. The proportion of screening conducted by each reviewer was further informed by the ‘Steps for Conducting a Scoping Review’.^28^ Firstly, all search results were imported to Zotero, where the duplicate records were removed. Secondly, the first author (ST) conducted screening on all articles while the second author (MM) screened 20% of the articles. Where an abstract was unclear or its relevance was uncertain, it was added for full-text screening. Thirdly, full text was screened; the first author (ST) screened all articles while the second author (MM) independently screened 50% of the articles. For all three stages (title, abstract, and full text screening), both authors (ST and MM) discussed any disagreements to reach a consensus. Prior to the first stage, the first and second authors (ST and MM) performed a calibration exercise to ensure consistent application of the eligibility criteria. A random sample of 10% of titles and abstracts was independently screened by each reviewer. A high level of agreement (94.4%) above the recommended target of 90% was achieved between the two authors (ST and MM). All disagreements were solved through discussion between the first and second authors (ST and MM). Lastly, the reference list of all included articles was checked for additional relevant citations. If the full-text version of an article was inaccessible, the original authors were contacted. An additional screening of the reference list of a recent scoping review on ‘unmet care needs of older people’^29^ identified five papers that were added to the list of full texts screened.^15,16,30–32^ The inclusion and exclusion criteria applied during the three stages (title, abstract, and full text) were defined following JBI's recommendations for scoping reviews (Table 2).^23^ All members of the review team are fluent in English. Additionally, the first author is also fluent in Mandarin, while the third, fourth and last authors are native Portuguese speakers.

**Table 2.** Data extraction guidance sheet.

Table entry	Description
First author; year; country	e.g., Brittain, K., 2010, United Kingdom. Use the year of publication of the article. Use the country where the study was conducted.
Aim	Record the specific objective(s) of the study.
Methods and instruments	Record the study design (e.g., qualitative, quantitative, mixed-methods), and instruments used for collecting data on unmet social needs (e.g., questionnaire, interview, etc.).
Participants	When available to include sample size, sex; age; ethnicity, education; socioeconomic status; marital status; carer relationship; living arrangements; cognitive assessments
Dementia type and stage	Identify the dementia type(s) and stage(s) involved in the study.
Findings on unmet social needs	When available to include contributing factors and consequences of unmet social need(s)).

### Data extraction

A spreadsheet for extraction was created using Microsoft Excel to retrieve data from the included studies. The first and second reviewers (ST and MM) each extracted data from 50% of the studies and cross-verified the data to ensure completeness and accuracy. Extracted data (where available) included: authors, year of publication and country; study aim(s), methods and instruments (study design, instruments used for collecting data on unmet social needs); participants (sample size, sex; age; ethnicity, education; socioeconomic status; marital status; carer relationship; living arrangements; cognitive assessments; dementia diagnosis method, type and stage); unmet social needs. The data extraction guidance sheet is provided in Table 2.

### Data analysis

Data were analysed using descriptive statistics for study characteristics and thematic analysis was conducted on qualitative results following the six-stage approach recommended by Braun and Clarke.^33^ Codes were developed inductively from the data, with themes generated from the reported experiences within the included studies through an iterative analytic process. The unit of analysis was the extracted qualitative data, which were coded sentence by sentence. The first reviewer (ST) familiarized with the data through repeated reading of the papers to gain a deep understanding of the content; systematically coded all relevant data in the papers; grouped related codes into potential themes; reviewed the themes against the entire coded data set; ensured themes were well-defined and finalized the themes; and presented the findings and themes according to the research questions. Throughout the process of thematic analysis, the first reviewer (ST) regularly shared and discussed the findings with the co-reviewers (LS, OR, and RMB). The final themes developed were discussed with the reviewers (LS, OR, and RMB) until a consensus was reached. A summary of the synthesized findings in a narrative form was provided. In line with the established guidelines,^23,24^ a quality rating of the studies and an assessment of the risk of bias were not conducted.

## Results

**Figure 1.**
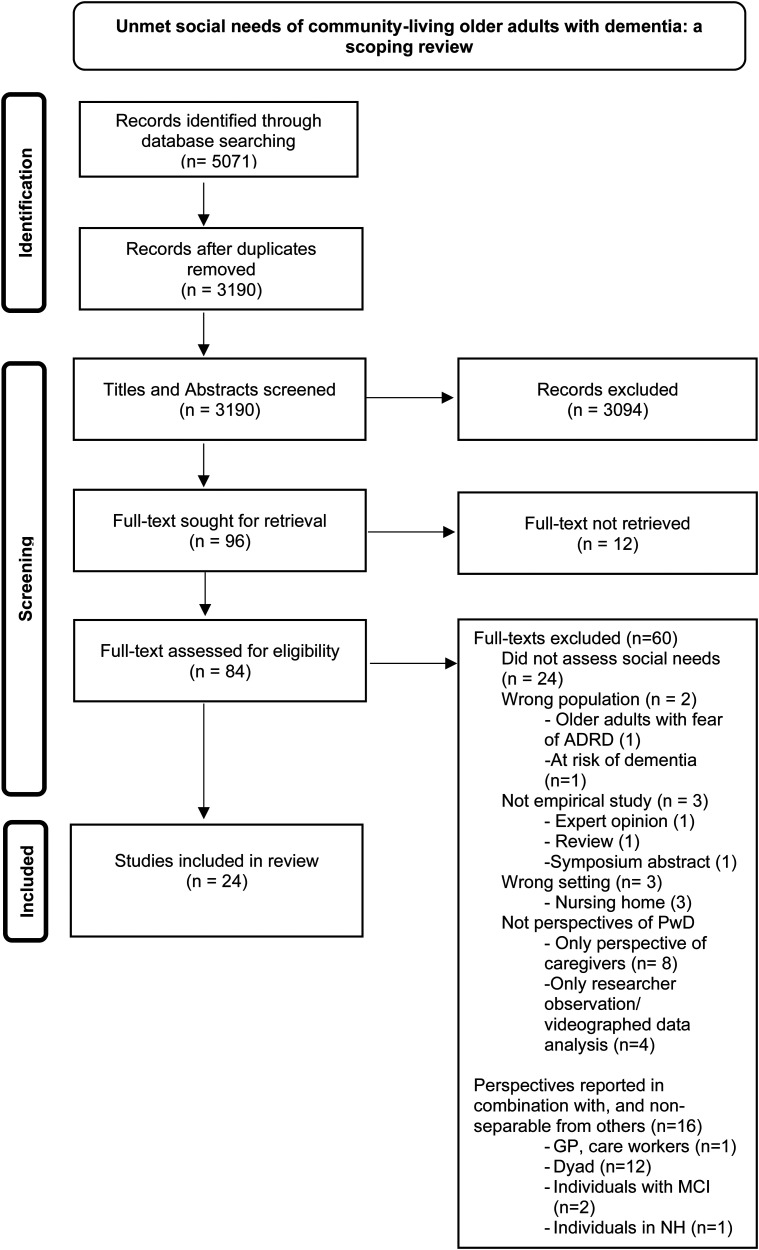
PRISMA 2020 flow diagram.

The search yielded 5071 records as potential articles for inclusion. After removing duplicates, 3190 articles were screened based on their titles and abstracts. One article^16^ was manually added through citation searching. After excluding irrelevant articles, 96 publications were retrieved for full-text assessment of eligibility. In total, 24 articles met the eligibility criteria and were included. The PRISMA-ScR flowchart detailing this process is presented in Figure 1. Detailed evidence regarding the included studies is presented in Table 3.

**Table 3.** Summary of the included studies.

First author, date & country	Aim(s)	Methods and instruments	Sample	Dementia stage and type
Brittain, K., 2010, United Kingdom^43^	Explore the varied meanings and lived experiences of older people with dementia, in relation to everyday technologies in public spaces outside the home.	Secondary analysis of qualitative data from two studies*.*	N = 16 people with dementia and 3 informal carers.	Stage: mild to moderate. Diagnostic method: not specified. Type: not specified.
Chung, P., 2019, United Kingdom^54^	To explore the user experiences of older people with dementia who had used homecare enablement to support transitions in daily life at home.	Qualitative. Semi-structured interviews.	N = 16 participants with dementia and 8 carers. Sex: 8 male, 8 female. Aged 70–90 years 8 carers: 3 male, 5 female; 5 spouses, 2 daughters-in-law, 1 son.	Stage: not specified. Diagnostic method: not specified. Types: Alzheimer's disease (AD), vascular-type and Lewy-body dementia.
Clare, L., 2022, United Kingdom^35^	To explore the impact of the pandemic on community-dwelling individuals with mild-to-moderate dementia and compare responses with pre-pandemic data.	Mixed-methods cross-sectional observational. Telephone or online interview. Open-ended and close questions.	N = 173 people with dementia and 242 carers (informants). Sex (PwD): 101 males (58.4%), 72 females (41.6%). Age: 74.33 years old (range 50–98). Education: no qualifications (20.8%), school leaving certificate at age 16 (18.5%), school leaving certificate at age 18 (37.6%), university (22.0%), missing (1.1%). Occupation: 12 Professionals (6.9%), 67 Managerial and technical (38.7%), 29 NM Skilled occupations – non-manual (16.8%), 32 M Skilled occupations – manual (18.5%), 20 Partly skilled (11.6%), 3 Unskilled (1.7%), 4 Armed forces (2.3%), 2 N/A (1.2%), 4 Missing (2.3%). Marital status: single (4.0%), married (50.9%), remarried (16.8%), civil partnership (1.2%), legally separated (1.2%), divorced (13.9%), widowed (8.7%), cohabiting (3.5%). Carer relationship: spouse/partner (86.8%), other family/friend (13.2%). Living: 21.4% alone, 74.6% with spouse/partner and 4% with other.	Type: Alzheimer's disease (AD) (45.1%), vascular dementia (VaD)(9.8%), mixed AD and VaD (15.0%), frontotemporal (13.9%), Parkinson's disease dementia (4.6%), Lewy bodies (8.1%), unspecified/other (3.5%) Diagnostic method :not specified, however 5-min MoCA and Estimated MMSE equivalent to MoCA score recorded. Stage: mild to moderate.
Darlington, N., 2021, United Kingdom^37^	To answer the questions: (1) Are people living with dementia aware of their local dementia-friendly community (DFC)? (2) Does awareness of a local DFC affect their experience of living with dementia? (3) What would help people living with dementia to live well in their local community? (4) Can survey methodology be used successfully among people living with dementia?	Cross-sectional survey. Survey over phone or mail. Completed independently or with support of carer.	N = 240 people with dementia. Sex: 106 males (46%), 126 females (54%). Eight not specified. Age: less than 64 (7.8%), 65–74 (25.1%), 75–84 (45%), 85+ (22.1%), not specified (9%). Ethnicity: White British/White other (95%), Black, Asian and minority ethnic (5%). Living arrangements: All community dwelling, living across 6 DFCs. 59 lived alone (23%), 174 lived with others (74.7%), 7 did not report (2.3%).	Stages: 127 early (57%), 72 middle (32.3%), 24 advanced (10.8%), 17 not specified (7.1%). Diagnostic method: not specified. Type: not specified.
Dawson, E., 2023, United Kingdom^36^	To explore how experiences of coping with the effects of the pandemic in the UK changed over time (after 22 months of social restrictions in England and Wales).	Mixed method. Qualitative semi- structured interviews.	N = 9 people with dementia Sex: 4 female, 5 male. Age range: 51 to 89 years old. Living arrangements: All living in the community. 5 lived alone, 4 lived with spouse/partner. Education: university degree (4), school leaving certificate at age 16 (2), professional qualification (1), vocational qualification (1), apprenticeship (1).	Type: Alzheimer's disease (5), frontotemporal dementia (2), posterior cortical atrophy (1), mixed dementia (1). Diagnostic method: not specified. Stages: Three to eight years since diagnosis of dementia. 3 years (1), 6 years (4), 7 years (3), 8 years (1).
Dickins, M., 2018, Australia^50^	To understand how different groups of individuals conceptualize the issue of risk for those living with dementia.	Qualitative. Semi-structured interviews.	N = 7 people with dementia. Sex: 3 females, 4 males. Mean age: 66 (S.D. 11.6).	Stages: Not specified. Mean years since diagnosis: 4.5 years (S.D. 2.2). Diagnostic method: not specified. Type: Not specified.
Eades, M., 2018, United Kingdom^44^	To trial the first intervention that brought cultural activities led by professional artists to isolated people with mild and moderate dementia in their own homes.	Qualitative. Open-ended Interviews.	N = 6 people with dementia, 4 artists, 3 befrienders Sex: 3 males, 3 females Age: Seventies (3), eighties (1), nineties (1). Living arrangements: Lived in own homes (4), sheltered accommodation (2).	Stage: Mild or moderate dementia (through inclusion criteria). Diagnostic method: not specified. Type: Not specified.
Eichler, T., 2016, Germany^51^	To describe the number and types of unmet needs of German primary care patients screened positive for dementia and factors associated with the number of unmet needs.	Cross-sectional analysis of randomized controlled trial. Interview based on standardised questionnaires.	N = 227 people with dementia. Sex: 121 females (53.3%), 106 males (46.7%). Mean age: 80.3 years old. Living arrangements: 115 lived alone (50.9%), 110 lived with others (49.1%).	Stage: From no impairment to severe impairment. Diagnostic method: screened by primary care practitioner to fulfil the International Classification of Diseases, Tenth Revision(ICD-10) and have a Demenz-Detektion-score < 9 (DemTect). Cognitive impairment (MMSE): 22.1 (5.3). No impairment 50 (22.9%), Mild impairment 109 (50.0%), Moderate impairment 54 (24.8%), Severe impairment 5 (2.3%). Type: No formal diagnosis of dementia 145 (63.9%), Alzheimer's disease 20 (8.8%), Vascular dementia 22 (9.7%), Dementia in Parkinson's disease 4 (1.8%), Unspecified dementia 47 (20.7%), Other degenerative diseases 6 (2.6%).
Flatt, J. D., 2015, USA^39^	To describe the subjective experiences of older adults with early-stage Alzheimer's disease or related cognitive disorders (ADRDs) and their family caregivers regarding an art museum engagement activity.	Qualitative. Focus groups.	N = 10 persons with ADRD and 10 family caregivers. Sex: 5 females (50%), 50% 5 males (50%). Age: 8 were >60 years. Ethnicity: Caucasian (7), African American (3). Dyads all had their own transportation to the museum and were physically able to participate in the walking part of the guided tour.	Stage: Early-stage Alzheimer's disease (8), Alzheimer's Disease related cognitive disorder (2). Diagnostic method: evaluation performed by the University of Pittsburgh Alzheimer's Disease Research Center Type: not specified.
Hicks, B., 2020, United Kingdom^52^	To address social exclusion among rural-dwelling older men with dementia in an English county.	Participatory Action Research (PAR). Focus groups, open interviews, reflexive field-notes.	N = 22 people with dementia, 15 care partners, 5 community volunteers. Sex: All male. Age : 68–90. Living arrangements: All community-dwelling. Main carer: Wife (18), lives alone (3), employs live-in helper (1) Ethnicity: All White British	Stage: 3 to 12 year since diagnosis. Diagnostic method: not specified. Type: not specified.
Manson, A., 2020, USA^45^	To describe perceptions of life satisfaction in early-to mid-stage Alzheimer's disease from both patient and caregiver perspectives.	Exploratory qualitative. Semi-structured dyadic interviews.	N = 4 PwD and caregivers. Sex: 3 female PwD/ male caregiver dyads, 1 male PwD/female caregiver dyad. Age: 55–80 years old. Living arrangement: All community dwelling, lives with caregiver. Ethnicity: All Caucasian non-Hispanic	Stage : Early-to mid-stage (mean MMSE = 22). Diagnostic method: not specified. Type: not specified. (recruited from a local Alzheimer's Association early-stage support group).
Mazurek, J., 2019, Poland^16^	To assess the needs of people with dementia living at home.	Cross-sectional study. Instruments: CANE, socio-demographic and QoL data.	N = 47 PwD, 41 informal carers. Sex: 34 females (72.3%), 13 males (27.7%). Mean age: 76.6 years (S.D. 13.3) Marital status: 24 Married (51.1%), 22 Widowed (46.8%), 1 Partner relationship (2.1%). Education: 15 Higher education (31.9%), 23 Vocational level 3 (48.9%), 4 Vocational level 2 (8.5%), 4 Qualification at level I or below (8.5%), 1 No qualifications (2.1%). Main carer: 23 Children (48.9%), 18 Spouse (38.3%).	Stage: Mild to moderate dementia (mean MMSE = 20.9 points +/-3.4) Diagnostic method: not specified. Type: not specified.
Miranda-Castillo, C., 2013, United Kingdom^18^	To compare perceived needs from the perspectives of people with dementia, family caregivers, and professionals.	Cross sectional survey. CANE interviews by psychologist and old age psychiatrists.	N = 125 PwD, 125 family caregivers. Mean age: 79.2 years (S.D. 6.8). Sex: 74 males (48.7%) and 78 females (51.3%). Ethnicity: White (98.7%), Black (0.7%), Asian (0.7%). First language: English (97.3%), Other (2.7%). Education: Higher education (20.3%), Secondary (75.6%, Below secondary (4.1%) Marital status: Single (2%), Married/living with partner (55.3%), Separated/divorced (4.6%), Widowed (38.2%). Living situation: Lives alone (32.9%), lives with others (67.1%) Has caregiver: Yes (90.1%), No (9.9%).	Stage: Mild to moderate (MMSE = 19.13 (S.D. 7.2) Diagnostic method: via the Diagnostic and Statistical Manual of Mental Disorders criteria (DSM-IV). Type: not specified.
Morrisby, C., 2019, Australia^48^	To identify support needs from the perspectives of people with dementia and their spousal carers.	Interpretive description study. Semi-structured dyadic interviews and two focus groups.	N = 10 dyads. Sex: Not specified. Assumed 4 males and 6 females, based on pseudonyms. Mean age: 75 years. Living arrangement: All community-living, cared for by a spouse.	Stage: moderate (FAST: ranged = 4- 6.4, median = 5). Diagnostic method: via the Functional Assessment Staging (FAST) by the researchers. Type: not specified.
Moyle, W., 2011, Australia^40^	To explore the perceptions of loneliness from the perspectives of people with early-stage dementia, living in community and long-term care settings	Descriptive exploratory qualitative study. Semi-structured interviews.	N = 70 PwD and 73 family carers. Sex: females (72.5%), males (27.5%). Age range: 66–97 years. Living arrangement: 60 LTC, 10 community.	Stage: Early stage Diagnostic method: not specified. Type: not specified.
Stapley, S., 2025, United Kingdom^38^	To assess what is important for ‘living well’ with mild-to-moderate dementia and how this changes over time.	Qualitative study. Semi-structured interviews, Sociograms as visual representations.	N = 20 PwD Sex: 10 Male, 10 female Age: 65- 86 years.	Type: Alzheimer's disease (n = 15), vascular dementia (n = 2), mixed dementia (n = 2), frontotemporal dementia (=1) Diagnostic method: clinical diagnosis by National Health Service memory services and other specialist clinics. Stage: Mild to moderate. (MMSE at 3 years prior to first interview in 2017): 22- 30; Time since diagnosis: Less than 1 year (n = 8), 1–2 years (n = 4), 3–5 years (n = 5), less than 3 years (n = 1), not available (n = 2))
Strandenæs, M. G., 2018, Norway^46^	To explore experiences of day care attendees’ living with dementia.	Qualitative descriptive study. Individual interviews, and interviews of next of kin.	N = 17 PwD Sex: 10 females, 7 males. Mean age: 80.5 years (range 72–92). Living arrangements: 9 lives alone, 8 lives with caregiver.	Stage : from very mild to moderate (Clinical Dementia Rating: Very mild (3), mild (11), moderate (3)). Diagnostic method : via the Clinical Dementia Rating Scale. Type : not specified.
Suwa, S., 2018, Japan^49^	To explore the experiences of a nonagenarian-centenarian woman with dementia living alone.	Qualitative content analysis of diary.	N = 1. Age: 106 years Sex: female. Living arrangement: Lives alone.	Type: Alzheimer's disease. Diagnostic method: by a physician, using the Hasegawa's Dementia Scale. Stage was severe dementia from 2010 to 2013.
Svanström, R., 2015, Sweden^53^	To elucidate the phenomenon of living alone with dementia and having a manifest care need.	Phenomenological interviews. Field notes and transcriptions from tape-recorded conversations were analysed.	N = 6 PwD Sex: 5 females, 1 male. Age: 80–90 years years. Living arrangement: All lived alone, had home care services on a daily basis, enrolled in day care services several days a week Has children: Yes (4), No (2).	Type: not specified Diagnostic method: not specified. Stage: not specified.
Tranvåg, O., 2015, Norway^47^	To explore and describe qualities of relational interactions preserving dignity among people with dementia, while interacting with family, social network, and healthcare professionals.	Qualitative research study. Interviews conducted with a modifiable interview guide.	N = 11 PwD Sex: 5 females, 6 males. Age: 64–85 years. Living arrangement: Lives with spouse in their own homes. Ethnicity: 8 native Norwegians, 2 born in other European countries, 1 was originally from Asia. The latter 3 had lived their adult lives in Norway.	Stage: mild to moderate. Diagnostic method: not specified. Type: Alzheimer's Disease (10),; mild cognitive impairment (1).
van der Roest, H. G., 2009, Netherlands^27^	To assess needs of community-dwelling people with dementia as reported by themselves and their informal carers.	Cross-sectional design.	N = 236 PwD and 322 informal carers Sex: 182 female (54.8%), 150 male (45.2%) Mean age: 79.8 (S.D. 7.6) years Marital status: Married (57.8%), Widowed (33.4%), Other (8.7%), Lived alone (36.5%) Education: 30 High level of education (12.7%), 6 Unknown (2.5%). Living arrangement: All community-living.	Stage: No cognitive decline (GDS 1) (n = 3, 1.3%), very mild cognitive decline (GDS 2) (n = 27, 11.4%), mild cognitive decline (GDS 3) (n = 51, 21.6%), moderate cognitive declined (GDS 4) (n = 76, 32.2%), moderately severe cognitive decline (GDS 5) (n = 44, 18.6%), severe cognitive decline (GDS 6) (n = 16, 6.8%), very severe cognitive decline (GDS 7) (Nil), severity unknown (n = 19, 8.1%). Diagnostic method: Global Deterioration Scale. Type: Alzheimer's disease (n = 91, 38.6%), vascular dementia (n = 33, 14.0%), mixed dementia (n = 29, 12.3%), other type (n = 22, 8.5%), type unknown (n = 63, 26.7%).
Van’t Leven, N., 2019, Netherlands^42^	To examine how dyadic interventions fit the needs, characteristics, and preferences of people with dementia and their informal caregivers.	Qualitative study. Semi-structured interviews.	N = 34 dyads, 19 professionals Sex: 22 males, 12 females Main carer: 28 partners, 6 parent/child.	Stage: Onset 1–5 years prior; average onset 2.4- 2.9 years. Diagnostic method: not specified. Type: not specified.
Wilkins, J. M., 2022, USA^41^	To examine values and preferences of community-dwelling persons with cognitive impairment and explore potential predictors of everyday preferences.	Cross-sectional study. Instrument: Preferences for Everyday Living Inventory (PELI).	N = 62 older adults with cognitive impairment Sex: males (57%), females (43%). Mean age: 78.9 years (S.D 7.1) Education: Mean 16.5 years (S.D. 2.8) Ethnicity: White (88.7%), Black or African American (6.5%), Asian (3.2%), American Indian or Alaska Native (1.6%) Marital status: Married/domestic partner (75.8%), Widowed (14.5%), Divorced (6.5%), Separated (1.6%), Never married (1.6%) Living arrangement: All community-living.	Type: Amnestic (76.9%), Lewy Body (7.7%), Non-amnestic (15.4%), MCI (35.5%), Cognitively Impaired, non-MCI (43.5%). Diagnostic method : by physician, using the Clinical Dementia Rating global score. Stage : not specified. (CDR Score : 0.5 (82.3%), 1 (17.7%). Mean MoCA score: 23.4 (S.D. 4.5))
Willis, R., 2020, Pakistan^34^	To understand perspective on help-seeking, understanding of diagnosis, stigma, and religion among people with dementia in Pakistan.	Exploratory qualitative study. Semi-structured interviews, with caregiver assistance where needed.	N = 20 PwD. Sex: 9 Females, 11 Males. Age: 40–49 (1), 50–59 (1), 60–69 (6), 70–79 (8), 80–89 (3), 90–99 (1). Living arrangements: all lives with caregiver in the community. Former occupation: Housewife (6), Business (3), Artist (1), Mosque administrator (1), Pharmacist (1), Teacher (2), University faculty (1), Contractor (1), Radio worker (1), Engineer (1), Not known (2).	Stage: mild stage. Diagnostic method : not specified. Type: not specified.

### Overview of the included studies

The included studies were published between 2009 to 2025, of which 19 (79%) were published since 2015. Nine studies were conducted in the United Kingdom (37%), three in the United States of America (12.5%), three in Australia (12.5%), two in the Netherlands (8.3%), two in Norway (8.3%), and a study each (4.1%) in Sweden, Germany, Poland, Pakistan and Japan. Nineteen studies (75%) used qualitative methods and five used quantitative methods (25%).

The included studies reported information on 1382 older adults with dementia. Twenty studies (83%) recruited participants aged ≥ 65 years, except for four studies which included participants of an age range of 40–99^34^; 50–98^35^; 51–89^36^; and younger than 64 years.^37^ 56% of participants were female. Three studies were part of the British IDEAL cohort study.^35,36,38^

The participants were in distinct dementia stages. Most studies involved participants who were in the earlier stages, with four specifically including participants in the mild stage^34,39–41^; ten studies included participants who were in mild to moderate stages.^16,18,35,38,42–47^ Two studies involved participants with moderate stage dementia,^36,48^ and one study included participants with severe-stage dementia.^49^ Five studies spanned multiple stages from mild to severe-stage dementia.^27,37,50–52^ Two studies did not specify the dementia stage.^53,54^

In terms of diagnosis, 12 studies provided specific information, reporting on a range of dementia types, including Alzheimer's disease, vascular dementia, mixed Alzheimer's disease, dementia with Lewy bodies, Parkinson's disease dementia, and frontotemporal dementia. The remaining 12 studies did not specify the type of dementia.

### Unmet social needs

Seven categories of unmet social needs were identified (Table 4), addressing the first research question. Each unmet social need is described in detail, based on the reported experiences from the included studies. The second research question aims to describe how unmet social needs vary across the dementia stages. Many studies did not include participants from a single stage of dementia, which limited our ability to draw stage-related trends. Variations across the stages of dementia are described based only on studies involving single stage participants (Table 4). In addition to stage-related patterns, the analysis also identified several cross-cutting influences that shaped how unmet social needs emerged and were experienced. Among these, stigma appears consistently as an embedded and transversal factor influencing social participation, help-seeking, and interpersonal relationships.

**Table 4.** Main findings from included studies.

		First author, date & country	Main findings
Unmet needs for participation in out-of-home activities	Outdoor social activities	Darlington et al., 2021, United Kingdom^37^	Reduced opportunities to engage in activities such work, meeting friends/ family, leisure activities, shopping etc.
		Strandenæs et al, 2018, Norway^46^	Participants felt that activities offered at day care does not fully match their personal preferences and interests.
		Svanström & Sundler, 2015, Sweden^53^	Participants began to lose engagement with activities that they previously liked and provided opportunities for social interaction.
		Eichler et al., 2016, Germany^51^	Participants indicated unmet needs for social activities.
		Stapley et al., 2025, United Kingdom^38^	Participants reported challenges in maintaining hobbies.
		Wilkins et al., 2022, USA^41^	Participants rated social engagement (e.g., family contact, meeting new people, volunteering) as significantly more important than other aspects of everyday living.
	Gaming	Hicks et al., 2020, United Kingdom^52^	Reduced options for games that promote enjoyment without the pressures to perform to a certain standard.
	Art-based activities	Flatt et al, 2015., USA^39^	Participant had concerns about not being able to actively participate in (art-based) activities, due to lack of more detailed descriptions of the activity.
	Cultural activities	Eades et al., 2018, United Kingdom^44^	Participants had reduced participation in cultural activities such as playing the guitar or being in a drama group.
	Opportunities to contribute to others	Darlington et al., 2021, United Kingdom^37^	Participants desired for activities such as being able to continue driving and be able to teach others.
		Wilkins et al., 2022, USA^41^	Participants rated social engagement (e.g., Family contact, meeting new people, volunteering) as significantly more important than other aspects of everyday living.
	Daytime activities	Miranda-Castillo et al., 2013, United Kingdom^18^	Participants reported unmet needs in the areas of ‘daytime activities’ in the Camberwell Assessment of Needs for the Elderly.
		Mazurek et al., 2019, Poland^16^	
Unmet need for support to maintain independence in daily life	At home	Chung, 2019, United Kingdom^54^	Participants want to use their abilities to keep doing what they can, such as keeping the house tidy, instead of solely depending on their family's help.
		Manson et al., 2020, USA^45^	Participants want to be able to remember their own appointments so that they do not impose on others.
		Suwa et al., 2018, Japan^49^	The participant wanted to be as independent as possible in living alone and even refusing care from family and care staff.
		Van’t Leven et al., 2019, Netherlands^42^	Participants did not find themselves to be self-sufficient in daily activities and required assistance from their caregivers.
	In public	Darlington et al., 2021, United Kingdom^37^	People with dementia have stopped one or more activities because of the lack of required support in public spaces.
		Svanström & Sundler, 2015, Sweden^53^	People with dementia reported feeling reluctant to leave the home alone due to not having support with unfamiliar environments.
		Brittain et al., 2010, United Kingdom^43^	Participants felt out of home activities have become increasingly constrained by the anxiety of family members.
		Stapley et al., 2025, United Kingdom^38^	Participants avoided going out on their own without their carers
	In work environment	Dickins et al., 2018, Australia^50^	Participants reported the feeling of isolation while they experience of loss of independence in activities, such as driving, working and finances. Participants expressed their feeling that they could have continued working if reasonable adjustments were made.
		Stapley et al., 2025, United Kingdom^38^	Participants reported that they missed work since their retirement and needed to keep themselves busy with activities.
	In religiosity	Willis et al., 2020, Pakistan^34^	Participants explained how difficulty with orientation in time interferes with knowing the correct time of day to offer prayers.
	In mobility and transportation	Moyle et al., 2011, Australia^40^	Participants reported mobility challenges impacting their participation in activities.
		Morrisby et al., 2019, Australia^48^	Participants found that the experience and transition of losing their driving ability was distressing.
		Manson et al., 2020, USA^45^	Participants reported changes in their ability to drive affecting their participation in activities.
		Darlington et al., 2021, United Kingdom^37^	Participants commonly reported that they do not continue to drive and felt there could be better public transport options.
Unmet need for companionship	Friends and family members	Moyle et al., 2011, Australia^40^	Participants desire for more visits from family. Participants felt the absence of family and friends particularly during certain times of day and occasions.
		Svanström & Sundler, 2015, Sweden^53^	Participants wish for more visits from family and friends for companionship and conversation.
		Suwa et al., 2018, Japan^49^	The participant's does not have visitors as she lives alone. Some of her family members lived a distance away, while others have passed away.
	Friends	Hicks et al., 2020, United Kingdom^52^	An individual described that he has difficulty feeling comfortable with being friends with others in the group. They are facing similar situations (dementia), and that is the only thing they have in common.
		Stapley et al., 2025, United Kingdom^38^	An individual described not having new friends, and wanting new friendships just like his wife is able to.
	Company	Miranda-Castillo et al., 2013, United Kingdom^18^	Participants reported unmet needs in the areas of ‘company’ in the Camberwell Assessment of Needs for the Elderly.
		Mazurek et al., 2019, Poland^16^	
		van der Roest et al., 2009, Netherlands^27^	
Unmet need for meaningful relationships	Maintenance of meaningful relationships	Moyle et al., 2011, Australia^40^	Participants reported the absence and loss of meaningful relationships.
		Svanström & Sundler, 2015, Sweden^53^	Participants losing contact with people who are not close to them.
	Social networks knowledge and understanding about dementia	Morrisby et al., 2019, Australia^48^	Participants perceived that people in their broader social networks such as work colleagues, lacked knowledge and understanding of dementia.
		Stapley et al, 2025, United Kingdom^38^	Participants perceived that people in their broader social networks such as friends, colleagues, lacked knowledge and understanding of dementia.
	Family understanding and acceptance of dementia	Suwa et al, 2018, Japan^49^	The participant felt that she was uninvited to her grandchild's wedding ceremony due to her dementia.
		Willis et al, 2020, Pakistan^34^	Some family members thought that the person with dementia was pretending.
	Family and friends understanding and acceptance of dementia	Moyle et al., 2011, Australia^40^	Participants report that family and friends could not understand, accept or come to terms with the changes in the person with dementia
	Maintenance of conjugal relationship	Stapley et al., 2025, United Kingdom^38^	An individual reported that behavioural changes strained their spousal relationships and increased dependence on their spouse for social interactions.
Unmet need for respectful and dignified social interactions	Public and community understanding	Darlington et al., 2021, United Kingdom^37^	People with dementia felt that better public knowledge of what it is like to live with dementia will help them to live well in their community. People with dementia desired living in a community free from stigma.
		Hicks et al., 2020, United Kingdom^52^	Participants faced uneasy tensions in a group, when there are conflicting interests, facing disparaging comments or others refusing to engage with them.
	Respect (privacy) regarding dementia diagnosis	Darlington et al., 2021, United Kingdom^37^	Participants did not want attention drawn to their dementia diagnosis.
		Morrisby et al., 2019, Australia^48^	A participant chose not to disclose their diagnosis and told acquaintances they had a diagnosis of Parkinson's disease.
	Respect in transition points	Morrisby et al., 2019, Australia^48^	Participants felt that there was a lack of respectful communication when they had to give up driving
	Respect of sense of masculinity	Hicks et al., 2020, United Kingdom^52^	A participant distanced himself from perceived vulnerability, after facing resistance from being integrated into a group, and felt that his sense of masculinity was threatened.
	Respect as fully capable individuals	Eades et al., 2018, United Kingdom^44^	Participants felt that they are not provided the same rights to be heard or fully participate in some activities.
	Respect from healthcare professionals	Tranvåg et al., 2015, Norway^47^	Participants reported healthcare professionals made them feel overlooked and devalued, not being acknowledged as an equal and making them feel small or unimportant.
		Chung, 2019, United Kingdom^54^	Some participants felt that home care staff failed to listen to participants’ viewpoints and were being ignored by staff who seemed to spend a lot of time writing reports rather than offering time for a meaningful conversation with them.
Unmet need for emotional support	From professionals during service provision	Morrisby et al., 2019, Australia^48^	Through the process from diagnosis to ongoing care participants perceived a lack of empathy from healthcare professionals, leaving them feeling unsupported, resulting in some turning to private healthcare.
	Regarding psychological distress	Suwa et al., 2018, Japan^49^	The participant experienced distress whenever the home helper who visits comes in daily, without permission.
		Miranda-Castillo et al., 2013, United Kingdom^18^	Participants reported unmet needs in the areas of ‘psychological distress’ in the Camberwell Assessment of Needs for the Elderly.
		Mazurek et al., 2019, Poland^16^	
		van der Roest et al., 2009, Netherlands^27^	
	During bereavement of a significant person	Moyle et al., 2011, Australia^40^	Participants were impacted by the loss of human connection and sharing after the death of someone close to them.
Unmet social needs during pandemic	Outdoor social activities	Dawson et al., 2023, United Kingdom^36^	Reduced participation in activities such as social events, exercise classes, travelling and substituting with indoor activities such as knitting, reading, and watching television. Participants also felt their communication skills and mental well-being declined when they did not have access to in-person dementia groups.
	Spouse companionship		Feeling distant from wife who now lives in a care home.
	Opportunities for meaningful conversations		Participants felt they lack meaningful conversations due to the closure of dementia advocacy and social groups.
	Emotional support from professionals		Participants felt like although they needed formal or professional emotional support, it does not exist. Participants felt the lack of social support that was once provided by the dementia groups (when the groups closed due to pandemic).
	Support from others to maintain independence in mobility and transportation	Clare et al., 2022, United Kingdom^35^	Participants reported difficulties with mobility.
	Companionship from family members		Participant felt that more family members were in contact compared to earlier during the pandemic. However, this did not directly translate to a higher satisfaction in family support.

### Unmet need for participation in out-of-home activities

The unmet need for participation in out-of-home activities was reported in eleven studies.^16,18,37–39^^,41,44,46,51–53^ It encompasses social, cultural, recreational, and outdoor activities such as meeting friends or family, attending events, or engaging in hobbies. These activities provide enjoyment, social connection, and a sense of purpose. This theme was described for the mild stage of dementia.^39,41^ Older adults with dementia desired art-based engagement; however, a lack of clear information about activities like museum visits was highlighted as a barrier to participation.^39^ Using the Preferences for Everyday Living Inventory (PELI), Wilkins et al.^41^ found that social engagement, including maintaining regular contact and volunteering, was highly valued yet often unfulfilled. This study's findings further revealed that social engagement was rated significantly more important than other everyday life domains, such as autonomous choice, personal growth, and maintaining a routine.

Studies which involved participants of mixed stages of dementia reported their desire to participate in out-of-home activities, yet barriers such as financial constraints and cognitive decline limit their involvement.^37,46,51^ Some participants reminisced about past cultural engagements or hobbies, which became challenging to maintain after dementia onset, a challenge further exacerbated by service and support closures during the COVID-19 pandemic.^36,38,44^ Among some older adults with dementia, activities that were once important, such as hobbies, engagement in clubs, and travelling, were described as becoming less meaningful and or enjoyable over time.^53^ Performance pressures during gaming activities was found to discourage participation among individuals with dementia.^52^ In two studies using the CANE instrument, ‘daytime activities’ was a significant unmet need - identified as the second most common unmet need in Miranda-Castillo et al.^18^ (14.5%) and the third most common in Mazurek et al.^16^ (25.5%).

### Unmet need for support to maintain independence in daily life

Twelve studies reported on the unmet need for support to maintain independence in daily living.^34,37,38,40,42,43,45,48–50^^,53,54^ This theme was described for the mild^34,40^ moderate^48^ and severe^49^ stages of dementia. In the mild stage, Moyle et al.^40^ reported that mobility challenges such as needing walkers or wheelchairs restricted their social participation. Willis et al.^34^ found that participants placed importance on maintaining independence in cultural routines such as religious practices. In the moderate stage, Morrisby et al.^48^ described how participants increasingly required support in daily activities due to reduced mobility and loss of driving privileges. Losing driving privileges was particularly distressing, especially when the decision was communicated by authorities or professionals in ways perceived as lacking sensitivity or respect.^48^ In the severe stage, a case study by Suwa et al.^49^ highlighted a participant's strong desire to live independently while living alone. Among studies which involved participants with mixed stages of dementia, needs for maintaining independence in daily life were identified across four areas: (i) their own homes, (ii) public spaces; (iii) workplaces, and (iv) mobility and transport. In their own homes, rather than solely relying on family support, many older adults with dementia wanted to continue engaging in household tasks to preserve their sense of self and purpose. Some resisted help when it was perceived as undermining their abilities, even if their families or carers felt it was necessary.^54^ Others acknowledged the need for assistance with specific activities, such as using household devices or managing routines, which reflected a need for balance between autonomy and support.^42,45^ In public spaces, a lack of support in public environments led some older adults to stop going out entirely, or avoid being on their own in unfamiliar settings.^37,38,53^ Additionally, family members’ concerns about safety often resulted in restrictions on independent mobility, contributing to reduced social participation and feelings of isolation.^43^ In workplaces, loss of employment was associated with a loss of identity and purpose.^38,50^ Some older adults with dementia felt they could have continued working if reasonable accommodations had been made, but instead faced abrupt restrictions following their diagnosis.^50^ In mobility and transport, some individuals limit themselves to familiar routes when leaving their homes.^37^

### Unmet need for companionship

Eight studies reported the unmet need for companionship.^16,18,27,38,40,49,52,53^ This includes regular contact with family, friends, or social groups, even if the interactions were casual or not deeply personal. Individuals expressed a desire for more visits from family and friends, frequent conversations, and opportunities to engage socially to avoid feeling isolated. This theme was described for the mild stage^40^ and severe^49^ stage of dementia. In the mild stage, Moyle et al.^40^ found that participants often felt the absence of regular interaction, especially during times such as holidays or nighttime. One participant described loneliness being most pronounced at night when not accompanied by his spouse.^40^ In the severe stage, Suwa et al.^49^ presented a case who described the emotional impact of living alone and having children who rarely visited.^49^ Among studies which included individuals with mixed stages of dementia, the need for companionship was not limited to family members, and the presence of any visitor regardless of their relationship provided a sense of meaning and connection.^53^ Some older adults with dementia felt isolated despite being part of dementia groups, suggesting that shared experiences of dementia did not necessarily lead to quality interactions.^52^ An individual reported that he would like to make new friends, and compared himself to his wife who could.^38^ In studies using the Camberwell Assessment of Need for the Elderly (CANE), the need for *company* was ranked the second^16^ and third^18,27^ most commonly reported unmet need cited by 29.8%, 12.8% and 5.4% of participants respectively.

### Unmet need for meaningful relationships

Six studies reported the unmet need for meaningful relationship.^34,38,40,48,49,53^ These studies highlighted the need for quality relationships that are emotionally fulfilling, which includes maintaining trusting and reciprocal relationships with close family, friends, and caregivers who are able to help the person living with dementia to feel understood, accepted and valued. This theme was described for the mild,^34,40^ moderate^48^ and severe^49^ stages of dementia. In the mild stage, Moyle et al.^40^ reported that family members did not understand the cognitive changes associated with dementia, sometimes perceiving memory lapses as intentional, leading to feelings of guilt and frustration. Similarly, Willis et al.^34^ found that family members were reported to suspect the older adult with dementia was “pretending” to forget, which strained family relations. In the moderate stage, participants felt that people in their broader social networks, such as colleagues, lacked an understanding of dementia, which contributed to feelings of being stigmatized.^48^ In the severe stage, an individual expressed sadness at not being invited to a grandchild's wedding, interpreting that she was left out due to her condition.^49^ Among studies involving participants with mixed stages of dementia, the perceived lack of empathy and understanding within families and friends often led to feelings of shame or being dismissed.^38^ One individual reported behavioural changes having a negative effect on their spousal relationship, and being more dependent on their spouse for social interactions.^38^ Others reported the lack of meaningful relationship due to the deterioration and challenges of maintaining close connections over time.^53^

### Unmet need for respectful and dignified social interaction

Six studies reported on the unmet need for respectful and dignified treatment.^37,44,47,48,52,54^ This includes unmet needs related to public knowledge and attitudes towards dementia, and the need to be treated with respect and dignity. The groups of people identified were families, friends, and broader social networks such as members of dementia support groups. Experiences reported include stigma, lack of understanding and acceptance, privacy of their diagnosis and difficulties in receiving the support they need. It includes situations where the person's voices and opinions were disregarded, leading to frustration and feeling undervalued. This theme was described for the moderate stage of dementia by one study,^48^ which highlighted a lack of respectful communication during transitions, such as during the process of losing the driver's licence; participants also felt that there is still a stigma associated with dementia that led some to choose not to disclose their diagnosis. Among studies involving individuals with mixed stages of dementia, community dwelling individuals perceived that the public needs a better understanding of what is it like to live with dementia.^37^ To avoid unwanted attention drawn to their diagnosis, some individuals choose to keep their diagnosis private.^37^ In a group of men who attended a game-based social support program, one individual faced disparaging comments and social exclusion.^52^ This lack of respect from the group made him feel that his sense of masculinity was under threat.^52^ Another topic within this theme regards the perception that the right to be heard and capacity to participate fully in activities were not respected.^44^ In their interactions with healthcare professionals, some participants felt that they were not acknowledged as an equal, resulting to them feeling overlooked and ignored.^47,54^

### Unmet need for emotional support

Six studies reported on the unmet need for emotional support.^16,18,27,40,48,49^ This theme focuses on needs related to the emotional and psychological well-being of older adults with dementia. Individuals reported needs for emotional and psychological support to engage in social situations, and cope with psychological distress or bereavement. This includes formal sources of emotional support from professionals. This theme was described for the mild,^40^ moderate^48^ and severe^49^ stages of dementia. In the mild stage, one participant described the loneliness experienced when reminiscing about shared experiences with a late loved one.^40^ In the moderate stage, Morrisby et al.^48^ reported a need for better emotional support during diagnosis and ongoing care. Some individuals perceived a lack of empathy from healthcare professionals, leaving them feeling dismissed or uncared for. One participant shared his disappointment with the memory clinic he attended, stating that some staff members “don't care”.^48^ In the severe stage, a participant experienced emotional distress when caregiving routines were perceived as intrusive, particularly when an assigned home helper entered the home without permission.^49^ In the studies involving individuals with mixed stages of dementia, psychological distress consistently emerged as the leading^16,18^ and third^27^ unmet need assessed with the CANE, indicating the prevalence of emotional support as an unmet social need.

### Unmet social needs during pandemic

Two studies reported unmet social needs that were related to the COVID-19 pandemic.^35,36^ Public health restrictions on social gatherings and the closure of community services led to increased social disconnection and isolation of older adults with dementia. Many individuals reported fewer opportunities for social interaction and meaningful conversation, contributing to feelings of isolation and decrease in emotional/psychological well-being. This theme was described for the mild stage of dementia by one study.^36^ The participants described the closure of dementia advocacy and social groups as particularly impactful. These groups had previously offered companionship, shared experience and advisors. The loss of such social connections due to the closure of groups left many feeling increasingly isolated. An additional factor contributing to social isolation reported in Dawson's et al.^36^ was the restriction of social gatherings beyond official guidelines, avoiding public places and events out of fear of harm to themselves or others. In place of group activities, some participants relied on solitary activities within the home such as knitting, reading, and watching television to remain occupied, although these did not fully replace the value of in-person connection. Over time, sustained isolation and lack of opportunities for social interactions led to marked declines in communication skills and mental well-being. As opportunities for social integration returned, several participants felt discomfort and frustrations when easing back into these social environments. Clare et al.^35^ involved individuals in mixed stages of dementia. While some participants observed an increase in family contact during the pandemic, this did not translate into stronger sense of emotional support or connection.^35^ In the same study, 39.3% reported difficulties in daily activities and 44.5% reported difficulties with mobility, both of which may limit their social participation.

### Variation in unmet social needs across dementia stages

Most studies included participant across multiple stages of dementia without distinguishing the specific unmet social needs in each stage. Therefore, stage-specific observations could only be drawn from a small number of studies (Table 5).

**Table 5.** Variation by stages.

Stage		Themes
Mild	Flatt et al., 2015^39^	Unmet needs for participation in out-of-home activities.
	Moyle et al., 2011^40^	Unmet need for support to maintain independence in daily life; companionship; meaningful relationships; and emotional support.
	Wilkins et al., 2022^41^	Unmet needs for participation in out-of-home activities.
	Willis et al., 2020^34^	Unmet need for support to maintain independence in daily life; and meaningful relationships.
Moderate	Morrisby et al., 2019^48^	Unmet need for support to maintain independence in daily life; and meaningful relationships; respectful and dignified interactions; and emotional support.
	Dawson et al., 2023^36^	Unmet social needs during pandemic.
Severe	Suwa et al., 2018^49^	Unmet need for support to maintain independence in daily life; companionship; meaningful relationships; and emotional support.

Results suggest that in the mild stage, unmet social needs tend to focus on maintaining activities and roles that reinforce personal identity, such as volunteering or community participation.^39,41^ Other unmet social needs observed include support for independence in daily life, companionship, meaningful relationships and emotional support.^34,40^ As dementia progresses into the moderate stage, unmet social needs shift towards navigating increasing dependence and dealing with role changes.^36,48^ Challenges related to acceptance from social networks emerge, along with more reliance on settings such as day centres, support groups, or home care services for social interactions.^36,48^ Emotional support remains essential in this stage.^48^ In the severe stage, changes in communication ability and reduced confidence in social settings further contributed to unmet needs for companionship, meaningful relationships and emotional support.^49^ These findings highlight a shift from actively seeking social engagement and autonomy in mild stages to requiring more supportive social interactions in the moderate and severe stages.

Beyond the life course trajectory of the older adult living with dementia, the family system's adaptation across the dementia stages also shapes unmet social needs. In the mild stages some families faced difficulties coming to terms with the condition, and even suspected the older adults to be “pretending” to forget.^34,40^ In the severe stages, role strains manifested in different ways ranging from overprotecting them and inevitably restrict their autonomy, to exclude them from family events.^49^ Families also experience their own unmet social needs, such as emotional support, respite and guidance from both informal networks and formal services.

### Stigma as a transversal influence on unmet social needs

Stigma was identified as an embedded factor shaping how unmet social needs emerged and were experienced across dementia stages, diverse cultural and national contexts. Older adults living with dementia reported feeling stigmatized, misunderstood or excluded^37,40,48,49^; as well as: ‘ignored by (healthcare) staff’,^54^ ‘wanting their right to be heard’,^44^ ‘negativity from family members’,^34^ ‘overlooked or devalued’.^47^ Studies reporting on stigma originated from diverse cultural contexts, including the United Kingdom, Australia, Norway, Pakistan and Japan, indicating that is a prevalent issue that transcends dementia stages and cultural contexts. Stigma was portrayed as a social experience that exacerbated unmet social needs. For instance, the fear of being judged or misunderstood led some individuals to withdraw from social interactions and hide their diagnosis, thereby further limiting opportunities for companionship, meaningful relationships and emotional support. This aligns with other literature showing that stigma towards dementia contributes to social exclusion, emotional withdrawal, reduced help-seeking and quality of relationships.^55,56^ This pattern leads to a vicious cycle where stigma contributes to isolation, and isolation in turn worsens stigma.^57,58^ Addressing stigma at the interpersonal, community and organizational socioecological levels is a necessary step in meeting the social needs of older people with dementia.

## Discussion

This scoping review synthesised evidence on unmet social needs among community-living older adults with dementia, with attention to how these needs evolve across the dementia trajectory. Existing literature on social participation, social engagement, and psychosocial well-being in dementia has largely examined individual perspectives or interpersonal relationships as separate domains. This scoping review extends understanding by conceptualising unmet social needs as multidimensional and relational, shaped by dynamic interactions across individual, interpersonal, and community contexts. Applying a socioecological framework offers a multilevel perspective on how unmet social needs emerge, are shaped, and evolve. It underscores the importance of supports that spans the multiple levels rather than focusing on individual factors.

### Multi-level influences on unmet social needs

**Figure 2.**
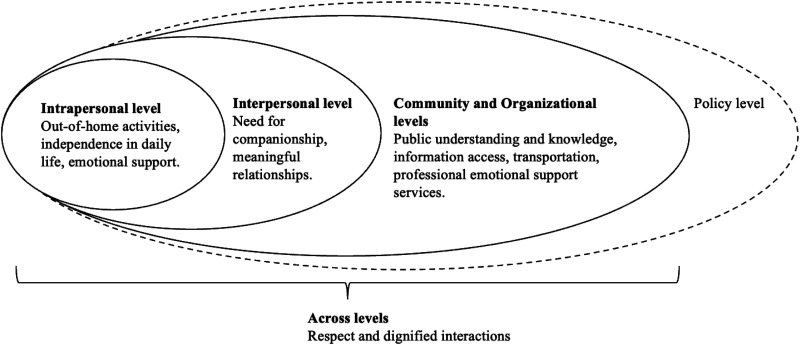
Unmet social needs across the socioecological levels.

While previous research has documented factors influencing social participation and engagement in dementia, it has generally examined these factors within a single domain, such as cognitive capabilities or caregiver characteristics, without considering how multiple levels interact to shape social experiences. Situating unmet social needs within a socioecological framework shifts the focus away from individual deficits toward modifiable relational, environmental, and structural influences. This perspective aligns with person-centred and rights-based approaches to dementia support,^59^ which emphasise inclusion, participation, and social citizenship. From an intervention perspective, these findings suggest that targeting unmet social needs should extend beyond the individual to coordinate efforts across families, services, and communities. Figure 2 applies a socioecological lens to the unmet social needs reported in the scoping review, emphasizing their multilevel nature.

### Implications for stage-responsive social support in dementia care

Although dementia is clinically recognized as a progressive condition, research exploring the unmet social needs and participation of people living with dementia has rarely adopted a stage-sensitive perspective. This review highlights both the relevance of dementia stage in shaping unmet social needs and the limited empirical attention given to stage-specific experiences. Most included studies involved participants across multiple stages of dementia, which restricted detailed comparisons, underscoring a gap in the existing literature. Despite these limitations, the synthesis suggests patterns that align with frameworks describing social and identity changes in people living with dementia. As dementia evolves, there is a gradual transition from autonomy-oriented unmet social needs toward increasing reliance on supportive and inclusive social environments.

These findings underscore the importance of regularly reassessing unmet social needs across the dementia trajectory, and recognizing the dynamic nature of preferences and capacities. Stage-responsive interventions may support continued engagement in meaningful activities and roles in earlier stages, while prioritising emotionally supportive, inclusive, and dignity-preserving social environments in later stages.

### Family coping as a contextual influence on unmet social needs

While caregivers, relatives and family members play a central role in shaping the social environments and opportunities of people living with dementia, family coping has rarely been examined as a contextual influence on unmet social needs. Existing research indicates that primary caregiver and family characteristics and behaviours can influence social participation outcomes.^60,61^ For example, partners of people living with dementia were considered as the most important social contact in reducing fear, and family members play key roles in initiating social activities.^60^ Caregivers and family members who are socially active have been found to promote greater social participation among people living with dementia.^61^ However, few studies explicitly explore how family adaptation, understanding, and coping processes shape the social experiences of people with dementia across stages.

Findings from this review suggest that family dynamics are closely intertwined with unmet social needs. In earlier stages, difficulties in family understanding and acceptance of the diagnosis sometimes led to misinterpretation of symptoms and strained relationships.^38^ In later stages, role strain manifested through overprotection or exclusion from family activities, inadvertently limiting autonomy and social inclusion.^49^ These patterns reinforce the view that unmet social needs are not solely a consequence of cognitive decline, but are also shaped by evolving family relationships and adaptive processes over time.

Literature has documented that caregiver needs and coping strategies vary across the stages of dementia, but are rarely examined in relation to the social participation and social needs of the person living with dementia. As a result, family coping is often treated as a static or background factor, limiting efforts to develop a more comprehensive understanding of unmet social needs. From an intervention perspective, these findings suggest that assessments and interventions aimed at addressing unmet social needs in dementia should incorporate caregiver, relatives and family coping and adaptation processes, recognising their potential to both support or constrain opportunities for social participation.

### Gaps in research

*Two main gaps were identified. First, scarcity of studies focusing on individuals in the moderate to severe stages of dementia.* Including participants across all stages of dementia, in particular in the moderate and severe stage, is essential for developing a more comprehensive understanding of the unmet social needs experienced throughout the dementia journey.

*Second, greater diversity in participant characteristics is needed*. There is a significant imbalance in geographical and socioeconomic representation of participants. For instance, there is a lack of research from countries with more family-oriented traditions such as in Southern Europe, where government and public policies support family-integrated approaches.^62^ The majority of included studies originate from higher-income and developed countries and lack exploration of how factors like community infrastructure and access to services may differ between urban and rural areas. It is equally important to include minority groups, which includes but is not limited to people with an immigrant background, sexual and gender diverse individuals, and individuals of diverse cultural backgrounds and social contexts. These populations may experience exclusions that affect their ability to have their social needs met. By actively engaging participants with a wide range of backgrounds, identities and lived experiences, future research can better capture the complexity and diversity of unmet social needs among older adults with dementia.

### Limitations

This scoping review has some limitations. First, it excluded studies published before year 2000, in languages other than English, Portuguese and Mandarin, and grey literature or reports from key organizations. However, independent screening by two reviewers and adherence to the JBI and PRISMA-ScR guidelines sought to reduce bias. Second, this review did not explicitly include the exploration of solutions to unmet social needs as a research question, which may have limited the identification of studies addressing such solutions. Third, differences in unmet social needs across dementia subtypes were not examined in this review as it was not a predefined objective. The lack of dementia subtype reporting and diagnostically mixed populations further limited the feasibility of subtype specific analysis. Nonetheless, existing literature on social and behavioural changes in neurodegenerative conditions suggests that unmet social needs may vary by diagnosis. The limited attention to dementia subtypes in research on social needs likely reflects a widely recognised methodological challenges in dementia research, including difficulties in recruiting sufficiently large and diagnostically homogeneous samples, overlapping symptom profiles, and the inclusion of participants across mixed disease stages. Addressing these challenges in future research may support the development of more diagnosis sensitive and stage responsive approaches to social support.

## Conclusion

This scoping review identified the following categories of unmet social needs: (i) participation in out-of-home activities, (ii) support for maintaining independence in daily life (iii) companionship, (iv) meaningful relationships, (v) respectful and dignified social interactions, (vi) emotional support, and (vii) social needs during the pandemic.

These needs evolve across dementia stages and are influenced by relational, socio-cultural, and structural contexts. The socioecological model provides a useful framework for understanding the multi-level factors that influence social needs. Future research should include a broader range of lived experiences, across all stages of dementia and reflecting diverse geographical, socioeconomic and cultural contexts. Greater inclusion of minority populations such as immigrants and sexual and gender diverse individuals is also essential. Strategies and approaches to addressing unmet social needs should be explored from the perspectives of older adults with dementia themselves.

## Supplemental Material

sj-docx-1-alz-10.1177_13872877261442966 - Supplemental material for Unmet social needs of community-living older adults with dementia: A scoping review Supplemental material, sj-docx-1-alz-10.1177_13872877261442966 for Unmet social needs of community-living older adults with dementia: A scoping review by Sunny Tan, Marine Markaryan, Óscar Ribeiro, Rita Maldonado Branco and Liliana Sousa in Journal of Alzheimer's Disease

## References

[1] World Health Organization. Decade of Healthy Ageing: Baseline Report. Summary. Geneva: World Health Organization, 2021.

[2] Bunt S Steverink N Olthof J , et al. Social frailty in older adults: a scoping review. Eur J Ageing 2017; 14: 323–334. 28936141 10.1007/s10433-017-0414-7 PMC5587459

[3] Steverink N Lindenberg S . Which social needs are important for subjective well-being? What happens to them with aging? Psychol Aging 2006; 21: 281–290. 16768575 10.1037/0882-7974.21.2.281

[4] Hartigan I Park G Timmons S , et al. Policy Position Paper: Dementia and Loneliness. Dublin, Ireland: Alzheimer Society of Ireland, 2019.

[5] Grand J Casper S MacDonald S . Clinical features and multidisciplinary approaches to dementia care. J Multidiscip Healthc 2011; 4: 125–147. 21655340 10.2147/JMDH.S17773 PMC3104685

[6] World Health Organization. Dementia Key Facts, https://www.who.int/en/news-room/fact-sheets/detail/dementia (2023).

[7] Warren A . Behavioral and psychological symptoms of dementia as a means of communication: considerations for reducing stigma and promoting person-centered care. Front Psychol 2022; 13: 875246. 35422728 10.3389/fpsyg.2022.875246 PMC9002111

[8] Feast A Moniz-Cook E Stoner C , et al. A systematic review of the relationship between behavioral and psychological symptoms (BPSD) and caregiver well-being. Int Psychogeriatr 2016; 28: 1761–1774. 27345942 10.1017/S1041610216000922

[9] Carvacho R Carrasco M Lorca MBF , et al. Met and unmet needs of dependent older people according to the camberwell assessment of need for the elderly (CANE): a scoping review. Rev Espanola Geriatr Gerontol 2021; 56: 225–235. 10.1016/j.regg.2021.02.004 33888307

[10] World Health Organization. ICD-11 for Mortality and Morbidity Statistics. Dementia. *International Classification of Diseases (ICD-11)*, https://icd.who.int/browse11/l-m/en#/http%3A%2F%2Fid.who.int%2Ficd%2Fentity%2F546689346 (2023).

[11] Costa S Sousa L Luz H , et al. Daily mobility and social interactions among community-dwelling older adults with pet dogs: a scoping review. J Appl Gerontol 2022; 41: 2609–2623. 36029015 10.1177/07334648221116633 PMC9669735

[12] Van Aerschot L Kadi S Rodrigues R , et al. Community-dwelling older adults and their informal carers call for more attention to psychosocial needs – interview study on unmet care needs in three European countries. Arch Gerontol Geriatr 2022; 101: 104672. 35279495 10.1016/j.archger.2022.104672

[13] Miranda-Castillo C Woods B Galboda K , et al. Unmet needs, quality of life and support networks of people with dementia living at home. Health Qual Life Outcomes 2010; 8: 132. 21073721 10.1186/1477-7525-8-132 PMC2995780

[14] Black BS Johnston D Rabins PV , et al. Unmet needs of community-residing persons with dementia and their informal caregivers: findings from the maximizing independence at home study. J Am Geriatr Soc 2013; 61: 2087–2095. 24479141 10.1111/jgs.12549 PMC4001885

[15] Black BS Johnston D Leoutsakos J , et al. Unmet needs in community-living persons with dementia are common, often non-medical and related to patient and caregiver characteristics. Int Psychogeriatr 2019; 31: 1643–1654. 30714564 10.1017/S1041610218002296 PMC6679825

[16] Mazurek J Szcześniak D Urbańska K , et al. Met and unmet care needs of older people with dementia living at home: personal and informal carers’ perspectives. Dementia (London) 2019; 18: 1963–1975. 28958171 10.1177/1471301217733233

[17] Janssen N Handels RL Sköldunger A , et al. Impact of untimely access to formal care on costs and quality of life in community dwelling people with dementia. J Alzheimers Dis 2018; 66: 1165–1174. 30400096 10.3233/JAD-180531

[18] Miranda-Castillo C Woods B Orrell M . The needs of people with dementia living at home from user, caregiver and professional perspectives: a cross-sectional survey. BMC Health Serv Res 2013; 13: 43. 23379786 10.1186/1472-6963-13-43 PMC3568411

[19] Morrisby C Joosten A Ciccarelli M . Do services meet the needs of people with dementia and carers living in the community? A scoping review of the international literature. Int Psychogeriatr 2018; 30: 5–14. 28784193 10.1017/S1041610217001491

[20] Curnow E Rush R Maciver D , et al. Exploring the needs of people with dementia living at home reported by people with dementia and informal caregivers: a systematic review and meta-analysis. Aging Ment Health 2021; 25: 397–407. 31791140 10.1080/13607863.2019.1695741

[21] Monin JK Jorgensen TD MacNeil Vroomen JL . Self-reports and caregivers’ proxy reports of unmet needs of persons with dementia: implications for both partners’ health-related quality of life. Am J Geriatr Psychiatry 2020; 28: 363–367. 31708379 10.1016/j.jagp.2019.10.006 PMC7089388

[22] Janssen N Handels RL Köhler S , et al. Profiles of met and unmet needs in people with dementia according to caregivers’ perspective: results from a European multicenter study. J Am Med Dir Assoc 2020; 21: 1609–1616.e1. 32674953 10.1016/j.jamda.2020.05.009

[23] Peters MDJ Marnie C Tricco AC , et al. Updated methodological guidance for the conduct of scoping reviews. JBI Evid Synth 2020; 18: 2119–2126. 33038124 10.11124/JBIES-20-00167

[24] Tricco AC Lillie E Zarin W , et al. PRISMA Extension for scoping reviews (PRISMA-ScR): checklist and explanation. Ann Intern Med 2018; 169: 467–473. 30178033 10.7326/M18-0850

[25] Peters MDJ Godfrey CM Khalil H , et al. Guidance for conducting systematic scoping reviews. Int J Evid Based Healthc 2015; 13: 141–146. 26134548 10.1097/XEB.0000000000000050

[26] Munn Z Peters MDJ Stern C , et al. Systematic review or scoping review? Guidance for authors when choosing between a systematic or scoping review approach. BMC Med Res Methodol 2018; 18: 143. 30453902 10.1186/s12874-018-0611-x PMC6245623

[27] van der Roest HG Meiland FJM Comijs HC , et al. What do community-dwelling people with dementia need? A survey of those who are known to care and welfare services. Int Psychogeriatr 2009; 21: 949–965. 19602305 10.1017/S1041610209990147

[28] Mak S Thomas A . Steps for conducting a scoping review. J Grad Med Educ 2022; 14: 565–567. 36274762 10.4300/JGME-D-22-00621.1 PMC9580325

[29] Kalánková D Stolt M Scott PA , et al. Unmet care needs of older people: a scoping review. Nurs Ethics 2021; 28: 149–178. 33000674 10.1177/0969733020948112

[30] Raivio M Eloniemi-Sulkava U Laakkonen M-L , et al. How do officially organized services meet the needs of elderly caregivers and their spouses with Alzheimer’s disease? Am J Alzheimers Dis Demen 2007; 22: 360–368. 10.1177/1533317507305178 PMC10846054 17959871

[31] Cohen-Mansfield J Dakheel-Ali M Marx MS , et al. Which unmet needs contribute to behavior problems in persons with advanced dementia? Psychiatry Res 2015; 228: 59–64. 25933478 10.1016/j.psychres.2015.03.043 PMC4451402

[32] Gaugler JE Kane RL Kane RA , et al. Unmet care needs and key outcomes in dementia. J Am Geriatr Soc 2005; 53: 2098–2105. 16398893 10.1111/j.1532-5415.2005.00495.x

[33] Braun V Clarke V . Thematic analysis: a practical guide. Los Angeles: Sage, 2022.

[34] Willis R Zaidi A Balouch S , et al. Experiences of people with dementia in Pakistan: help-seeking, understanding, stigma, and religion. Gerontologist 2020; 60: 145–154. 30452635 10.1093/geront/gny143

[35] Clare L Martyr A Gamble LD , et al. Impact of COVID-19 on ‘living well’ with mild-to-moderate dementia in the community: findings from the IDEAL cohort. J Alzheimers Dis 2022; 85: 921–936. 10.3233/JAD-215095 34776448

[36] Dawson E Collins R Pentecost C , et al. Navigating the coronavirus pandemic 2 years on: experiences of people with dementia from the British IDEAL cohort. Dementia (London) 2023; 22: 760–782. 36827539 10.1177/14713012231158215 PMC9969185

[37] Darlington N Arthur A Woodward M , et al. A survey of the experience of living with dementia in a dementia-friendly community. Dementia (London) 2021; 20: 1711–1722. 33031000 10.1177/1471301220965552 PMC8216308

[38] Stapley S Pentecost C Hillman A , et al. Continuity, change and ‘living well’ for older people with dementia: longitudinal qualitative findings from the IDEAL cohort study. Ageing Soc 2025; 45: 2187–2206. 40630177 10.1017/S0144686X24000333 PMC7617875

[39] Flatt JD Liptak A Oakley MA , et al. Subjective experiences of an art museum engagement activity for persons with early-stage Alzheimer’s disease and their family caregivers. Am J Alzheimers Dis Other Demen 2015; 30: 380–389. 25216658 10.1177/1533317514549953 PMC4362745

[40] Moyle W Kellett U Ballantyne A , et al. Dementia and loneliness: an Australian perspective. J Clin Nurs 2011; 20: 1445–1453. 21366740 10.1111/j.1365-2702.2010.03549.x

[41] Wilkins JM Locascio JJ Gunther JM , et al. Predictors of the importance of everyday preferences for older adults with cognitive impairment. Int Psychogeriatr 2022; 34: 287–294. 33455605 10.1017/S1041610220003956 PMC8286271

[42] Van’t Leven N de Lange J Prick A-E , et al. How do activating interventions fit the personal needs, characteristics and preferences of people with dementia living in the community and their informal caregivers? Dementia (London) 2019; 18: 157–177. 27509919 10.1177/1471301216662378

[43] Brittain K Corner L Robinson L , et al. Ageing in place and technologies of place: the lived experience of people with dementia in changing social, physical and technological environments. Sociol Health Illn 2010; 32: 272–287. 20003041 10.1111/j.1467-9566.2009.01203.x

[44] Eades M Lord K Cooper C . Festival in a box’: development and qualitative evaluation of an outreach programme to engage socially isolated people with dementia. Dementia 2018; 17: 896–908. 27466377 10.1177/1471301216658158

[45] Manson A Ciro C Williams KN , et al. Identity and perceptions of quality of life in Alzheimer’s disease. Appl Nurs Res 2020; 52: 151225. 31899042 10.1016/j.apnr.2019.151225

[46] Strandenæs MG Lund A Rokstad AMM . Experiences of attending day care services designed for people with dementia–a qualitative study with individual interviews. Aging Ment Health 2018; 22: 764–772. 28345965 10.1080/13607863.2017.1304892

[47] Tranvåg O Petersen KA Nåden D . Relational interactions preserving dignity experience: perceptions of persons living with dementia. Nurs Ethics 2015; 22: 577–593. 25319119 10.1177/0969733014549882

[48] Morrisby C Joosten A Ciccarelli M . Needs of people with dementia and their spousal carers: a study of those living in the community. Australas J Ageing 2019; 38: e43–e49. 10.1111/ajag.12609 30663189

[49] Suwa S Otani S Tsujimura M , et al. The diary of a nonagenarian-centenarian woman with dementia: memory loss, life changes, and community care in Japan. Int J Nurs Pract 2018; 24: e12655. 10.1111/ijn.12655 29667314

[50] Dickins M Goeman D O’Keefe F , et al. Understanding the conceptualisation of risk in the context of community dementia care. Soc Sci Med 2018; 208: 72–79. 29772396 10.1016/j.socscimed.2018.05.018

[51] Eichler T René Thyrian J Hertel J , et al. Unmet needs of community-dwelling primary care patients with dementia in Germany: prevalence and correlates. J Alzheimers Dis 2016; 51: 847–855. 26890767 10.3233/JAD-150935

[52] Hicks B Innes A Nyman SR . Exploring the ‘active mechanisms’ for engaging rural-dwelling older men with dementia in a community technological initiative. Ageing Soc 2020; 40: 1906–1938.

[53] Svanström R Sundler AJ . Gradually losing one’s foothold – A fragmented existence when living alone with dementia. Dementia (London) 2015; 14: 145–163. 24339094 10.1177/1471301213494510

[54] Chung P . Experiences of older people with dementia: homecare enablement to support transitions in daily life at home. Br J Occup Ther 2019; 82: 716–725.

[55] Brigiano M Calabrese L Chirico I , et al. Within my walls, i escape being underestimated: a systematic review and thematic synthesis of stigma and help-seeking in dementia. Behav Sci 2025; 15: 774. 40564556 10.3390/bs15060774 PMC12189192

[56] Patterson KM Clarke C Wolverson EL , et al. Through the eyes of others – the social experiences of people with dementia: a systematic literature review and synthesis. Int Psychogeriatr 2018; 30: 791–805. 29970210 10.1017/S1041610216002374

[57] Burgener SC Buckwalter K Perkhounkova Y , et al. The effects of perceived stigma on quality of life outcomes in persons with early-stage dementia: longitudinal findings: part 2. Dementia (London) 2015; 14: 609–632. 24339117 10.1177/1471301213504202

[58] Cohen-Mansfield J . The association between loneliness, social isolation and dementia – what does it mean? Int Psychogeriatr 2025; 37: 100003. 40086914 10.1016/j.inpsyc.2024.100003

[59] Smith SK Wolverson EL Mountain GA . What is intended by the term “participation” and what does it mean to people living with dementia? A conceptual overview and directions for future research. Front Rehabil Sci 2022; 3: 952722. 36189033 10.3389/fresc.2022.952722 PMC9397697

[60] Knecht HL Rodriguez FS . Social environment of people living with dementia: characteristics, facilitators and barriers. Alzheimers Dement 2024; 20: e087923.

[61] Palaza A Bouldin ED Miyawaki CE , et al. Characteristics of informal caregivers and social participation of people with dementia. Gerontologist 2024; 64: gnae096. 10.1093/geront/gnae096 PMC11398914 39093696

[62] Lillo-Crespo M Riquelme J Macrae R , et al. Experiences of advanced dementia care in seven European countries: implications for educating the workforce. Glob Health Action 2018; 11: 1478686. 30099937 10.1080/16549716.2018.1478686 PMC6095026

