# Supplementary material for: Unmet social needs of community-living older adults with dementia: A scoping review
Source: J Alzheimers Dis. 2026 Apr 23;111(4):1413–38. doi: 10.1177/13872877261442966 (PMC13234317; doi:10.1177/13872877261442966)
Supplement: sj-docx-1-alz-10.1177_13872877261442966 - Supplemental material for Unmet social needs of community-living older adults with dementia: A scoping review [file sj-docx-1-alz-10.1177_13872877261442966.docx]

**Supplemental Material**

**Unmet social needs of community-living older adults with dementia: A scoping review**

**Supplemental Table 1.** Search strategy for PubMed

|  | Query |
| --- | --- |
| #1 | "older people" OR elderly OR "old person" OR "older adults" |
| #2 | dementia OR Alzheimer OR "Neurocognitive disorder" |
| #3 | "social needs" OR leisure OR occupation OR interact* OR activit* |
| #4 | home OR community OR “community-living” |
| #5 | #1 AND #2 AND #3 AND #4  Limits:  - Abs/ title  - After year 2000  - Only Review, Clinical Trial, Randomized Controlled Trial  - Only English, Portuguese, Chinese language |

**Supplemental Table 2.** Extracted quotes from included studies

| **First author, date & country** | **Original quotes from article (without interpretation)** |
| --- | --- |
| Brittain, K., 2010, United Kingdom | - Everyday activities .. become increasingly constrained either by their own anxieties or by those around them.  - ...used to go out but his family were anxious for him not to (go out) any more… without the support of friends he would be confined inside more often.  - Well the family really, they’re frightened, you know, but I’m not, I’m not…(afraid to go out).  - Sometimes … panic attacks, if all of a sudden there’s a crowd of people,…I think ‘oh I don’t want to be here’…I will just go home and I’ll come back another time.  - I must admit my confidence has gone a bit, I used to love to go to [place], that’s my favourite place in the whole world.., but I haven’t been for a couple of years. |
| Chung, 2019, United Kingdom | - … wished to keep away from becoming a passive care-recipient to their family.  - ..for many (participants), with increasing risk and fear of falling, they lost confidence in their functional mobility and gradually adopted a sedentary lifestyle to avoid trips and falls.  - everything’s (in the house is) in a mess. My children had been here to tidy up the mess because they think I need help. But, the mess will never go away because I am the one who creates the mess. I mean, I have to use my abilities and keep doing what I can to stay sane and not screaming.  - Participants were keen to maintain their sense of self, reciprocity and being able to continually engage in daily activities as far as practical...seek for cooperation and interdependent relationships to support them at home...engaging in meaningful ‘doing’, within a trusting relationship.  - ‘enablement for myself. . .means being capable of coping with most things in life yourself. But also having the sort of mind where you don’t, and having the right people around you, that’s important’.  - Participants’ motivation to engage with the services was negatively influenced by staff approach when they failed to listen to participants’ viewpoints and to enable them to understand their care plan. Some felt they were being ignored by staff who seemed to spend a lot of time writing reports rather than offering time for a meaningful conversation with them. |
| Clare, L., 2022, United Kingdom | - 39.3% of PwD reported difficulty with everyday activities.  - 44.5% reported problems with mobility.  - ...more family members were in contact since IDEAL T3, but not the finding that people with dementia tend to be more satisfied with support from family. |
| Darlington, N., 2021, United Kingdom | - Participants still enjoy and engage in activities that require leaving the house. Example of activities are: going to work, meeting friends/ family, leisure activities, shopping etc.  - (Out of 240 participants)..fifteen respondents took park in group activities such as singing, gardening, bingo and a film club. Ten people were involved in sporting activities (cycling, walking and watching football), with only one sporting activity relying on the involvement of other people(dancing).  - 49 (20%) selected larger choice of enjoyable activities.  - 102 (43%) participants responded that more public understanding of what it is like to live with dementia would help them to live well in their community.  - One hundred and forty-eight (61%) respondents said that they had stopped one or more activities because of dementia. 52 (22%) selected extra support in public spaces, with 21% stating that they no longer went out alone.  - Of these, driving was the most frequently reported activity no longer undertaken (32%), 22 (9%) selected better public transport.  - Many of them were alternative expressions of the need to be free from stigma, to maintain their independence, and to feel they had a purpose and could still contribute (E.g., to continue driving and to teach others).  - There were a small number (n = 4) of responses that indicated the respondents did not personally want anything or did not want attention drawn to their dementia. |
| Dawson, E., 2023, United Kingdom | - (participants) limiting their meaningful activities, such as attending social events, exercise classes, travelling, and isolating themselves from society to protect themselves and others from potential harm.  - ... were imposing additional restrictions over and above those required by law, such as not going out and avoiding crowds, and relying instead on solitary activities within the home such as knitting, reading, and watching television to remain occupied.  - the impact of sustained isolation and limited stimulation had, perhaps unsurprisingly, led to a marked decline in mental wellbeing for some, most notably in those aged 81-90 who lived alone: “I don’t have any energy or enthusiasm to pick up a hobby of any sort.  - (participants became) increasingly isolated and lonely.  - (due to) limited stimulation, (becoming) apethetic; routine based solely on visiting and calling wife, who now lives in a care home.  - In-person dementia groups are closed... (leading to) decline in communication skills.  - (becoming) increasingly isolated, lacking meaningful converations.  -Dementia advocacy and social groups permanently closed, (leading to) lost contact with peers and advisors.  - To address this observed decline and worsening emotional wellbeing, one participant thought support from someone professionally trained for himself and other people with dementia would be useful: “Even though they (people with dementia) still need it just as much as anybody else…it doesn’t exist.  - ...one participant who was socially withdrawing due to age, (felt) meaningful, home-based activities such as woodwork became more difficult: “I do have to be careful what I’m doing now, because my brain no longer works in the same way that it used to”.  - ...notable losses were in communication skills as opportunities for social integration returned, highlighting the scale of the change to both themselves and others. For some, this decline has left them feeling “quite awkward with people,” reaching a point where “I can’t talk anymore. I can’t take it in anymore”.  -... is like a vicious downward spiral, isn’t it? Because you’re practising it less, you become even worse at it. And therefore, you avoid people more and more, and the level of isolation gets bigger and bigger.  - Several of these participants attended various virtual and in-person groups, appreciating the social, emotional and practical support they offered.  - Several participants were not receiving any social support for their dementia at this stage of the pandemic, instead demonstrating signs of social withdrawal: “I can’t do with socialising. No, people get on my nerves” |
| Dickins, M., 2018, Australia | -People living with dementia emphasised the experience of loss of independence, such as driving, working and finances. In particular, the experience of isolation and responsive behaviours were also highly salient to this group.  - People with dementia in particular spoke of the negative ramifications and often unintended consequences relating to the management of their cognitive and functional decline. For some they could see that activities or chores had been taken from them or redone leaving the person with dementia with a sense of purposelessness.  - People living with dementia spoke of the restrictions put in place when the diagnosis of dementia was given; for many it meant that activities they had done their whole lives were taken away from them overnight; I was 50 then, I lost my job as well and, in retrospect now, I know that if they'd made reasonable adjustments, I could have kept my job. |
| Eades, M., 2018, United Kingdom | - Participants reminisced about their cultural activities before dementia onset.  - I was never frightened of standing up and projecting when most people were embarrassed.  - I didn’t perform as showman. I didn’t perform but I played guitar.  - I was [shy] at first but from being in a drama group you get to open out. That’s a good thing. If you know a good drama group, you join.  - In other cases, the visits provided an opportunity to reflect upon the loss of cultural activity and other losses related to illness or old age.  -Participants often discussed their right to be heard and their capacity to participate fully in the activities.  - You’re asking the question, let me finish it.  - You see I’m not like most of your patients who are incapable. I’m capable of doing absolutely everything. |
| Eichler, T., 2016, Germany | - Among 227 primary care patients screened positive for dementia, 51 indicated unmet needs for 'social support/social activities'. |
| Flatt, J. D., 2015, USA | - People with Alzheimer’s you know you can be a part of art and be part of the process. Mildly impaired or even more impaired, you know you can have an opinion if you like something or not, so it’s a way to involve a lot of different . . . art touches a lot of different people, you can even express themselves.  - …more detailed description of the activities may have eased some of the participants’ reservations about participating; and it might help to minimize concerns about the inability to actively participate.  - It’s the small-group setting that’s definitely a plus; as opposed to a big group where there is a lot of confusion, information can’t be processed real well, so it’s definitely in the best interest to have small, intimate groups. |
| Hicks, B., 2020, United Kingdom | - ..on the rare occasions the men felt a sense of pressure to perform, the games lost their appeal. These instances usually occurred when the games were linked to activities the men were once competent with in their younger years, and as such they perceived a requirement to perform to a certain standard.  - The ‘diversity’ of the group and the ‘certain attitudes or interests’ became prominent, thereby making it difficult for certain men to ‘gel’. For instance in the Done Roaming group the tensions often centred on Doug, who felt his recent diagnosis of dementia was a threat to his masculinity. As such he sought to ‘other’ the rest of the men in the group, refusing to sit with them at the beginning of the early sessions of the initiative and instead watch from a quiet corner of the venue where he could talk to the volunteer.  - ‘Well, I found them a bit odd actually (other men in the group), but then I'm a Londoner. So I did find it very hard to become a friend of…an associate. Alright, they're there and they're in the same poo as you are, so that's about all it amounts to. I wouldn't want to visit them or go out with them. I don't know.’  - Within the Marching On group the men’s conflicting interests were more difficult to manage and resulted in a few men making disparaging comments to others or refusing to engage with them.  - For instance, Tel would roll his eyes when Chris spoke of his love for classical music and vice versa when Tel would speak of his days as a boxing coach. At one point after Chris played a classical piece of music on the piano, Dave exclaimed ‘yeah I like music, but none of that rubbish’. Others such as Jess also commented ‘oh ladeda, very posh!’ when Harry spoke at length about his home in the country and the tennis courts and horses he owned. This made for noticeable and uneasy tensions in the group that were only truly resolved when these men left. |
| Manson, A., 2020, USA | - I'd been doing everything that I thought I could do. I could drive, I could do this and I could do that and then all of a sudden it's not… I wish that I didn't have to be so needy. And the really, really, really, I don't like it. It's, I need to be me.  - I want to be able to remember those things (appointments) so I don't interrupt their workload and things like that. I don't want to be causing problems.  - And, and it's still, you know, every time when I get into the car I don't drive very far. But yet I enjoy being able to do it (driving). But since I just go mainly places that are well known, I just make sure I'm doing it the way a good boy should. |
| Mazurek, J., 2019, Poland | - The most common unmet needs according to the people with dementia were: psychological distress (31.9%), company (29.8%) and daytime activities (25.5%). |
|  |  |
| Miranda-Castillo, C., 2013, United Kingdom | - People with dementia reported fewer needs compared with the reports of their caregivers and the professionals. The most frequent unmet needs reported by people with dementia, were in the areas of daytime activities (14.5%), company (12.8%), and psychological distress (21.6%). |
| Morrisby, C., 2018, Australia | - (Regarding having a drivers license) ... [neurologist] said you have to let the transport people know, which I did. Mind you, within 10 days there was someone on our doorstep wanting the licence, which was a bit rude I thought.  - Knowledge and understanding of dementia among broader social networks, such as work colleagues, were described as a need at all stages of the disease.  - Many participants reported limited knowledge of dementia among others, or felt there was a stigma attached to the syndrome.  - One person with early-stage dementia preferred not to disclose their diagnosis and instead initially told acquaintances they had a diagnosis of Parkinson's disease.  - Many participants identified unmet needs during the process, from diagnosis to ongoing care. People with dementia and their carers identified a lack of empathy from health professionals and inconsistency in the care provided; for example inadequate referral to support services. Allen, a person with dementia, commented on his experience of diagnosis: “some people don't care [referring to memory clinic].” Three participants resorted to private health services to get the quality of care they sought. |
| Moyle, W., 2011, Australia | - Interviews revealed an association between the absence (and maintenance) of meaningful relationships and the experience of loneliness.  -There were examples of a desire for more visitors, or more generally, a desire for human contact and ‘people to talk to’ I would like more people to come to see us. That would be nice.  - My daughter doesn’t come to see me; none of my children come to see me. Sometimes I would like to talk to one or other of them.  - Family and friends lived some distance away and did not have the opportunity to visit.  -Loss of relationships was predominant in the theme staying connected to others. This was found to be especially apparent after the death of someone close to the person with dementia as this reduced the opportunity for human connection and sharing,  -...she knew every programme especially the cooking... but now she is not here and when [cooking show] comes on I say ‘look’, [but] she is not here, or I will say ‘look he has that meal on again’ or something that relates on the TV relates to something we used to do together or watch together. I will say ‘here is that music show we like’ or ‘here is so and so’ this is when I find I am lonely.  -reduced mobility or being confined to a wheelchair or bed greatly impacted the ability of people with dementia to have social interactions and participate in activities, as expressed by the following participants: I’ve shut myself away too much. I have to walk with a walker...There are a few of us with walkers so we don’t get out you know.  -... family and friends not accepting the diagnosis or not coming to terms with the changes occurring in the person with dementia as the illness progressed.  - Sometimes it can be hard, they don’t seem to think it should be lost, they think I should be able to remember exactly what you want to. Some say that, so I just say I try that is all I can do.  -...reported great shame when signs of memory loss were displayed. Two participants explained the personal impact of not remembering names, or recognising faces.  - Especially peoples’ names, people I have known for a long time and I can’t think of their names...  - Increased feelings of loneliness at certain times of the day/ year  -Time was identified as a factor in the experience of loneliness in people with dementia. Loneliness was found to be temporal in that people with dementia differentially experienced it depending on the time of the day.  -If I feel lonely, it’s at night when I miss my wife.  - Participants also referred to certain times of the year such as Christmas, Easter and Birthdays when the experience of loneliness was more pronounced. |
| Stapley, S., 2025, United Kingdom | - Fred missed work since retirement and needed to keep busy with activities every day: ‘once I lose them things, I get so miserable’ (Fred: T1).  - For Grace, giving up dancing when local classes stopped, her only interest and when she had been happiest, was a huge loss for her: ‘  -  I feel inadequate, I suppose. Um, because I can’t do some of the things that I always used to do and never ever thought about. Um, so that’s… that’s my grievance with this’ (Pam: T2).  - Lose self-confidence, it was not always clear where this had stemmed from, that is, whether from worsening symptoms or co-morbidities or perhaps from social expectations of what people with dementia are able to do.  - Jen and Sarah had been irritated by the dismissive reaction to their condition on disclosing this to other older people who, perhaps to ‘normalise’ dementia, had said that they also had memory problems:  - (due to) aphasia and hence found it difficult to engage socially: ‘I do sometimes feel left out for that reason… I’m not noticed’  - Terry wanted to forge new friendships himself, having noticed his wife’s friendships and wanting something similar for himself: ‘I haven’t got anyone saying you’re my friend  - ‘it’s a little part of our relationship that’s chipped out the whole lot’ (Pam: T2).  - increasingly dependent on their spouses for their social interaction; one was hoping her husband would retire so they could spend more time together  - lost’ without his wife and ‘terrified’ of going anywhere without her: ‘I’m not allowed out on my own’  - Tom’s wife was about to go on holiday without him |
| Strandenæs, M. G., 2018, Norway | - Some of the participants stated that there were activities they did not appreciate as much, but they saw them as a part  of the centre's arrangements. They felt free to participate as much as they liked, so if they did not appreciate the activity,  they could simply withdraw from it and observe the activities from a distance.  - ...they could not expect to be offered more of those activities that they really enjoyed, such as concerts or a trip to the woods with all of the others. They explained that these types of activities had a cost for the day care and that the cost was too high for the participants to expect to do more of these activities.  - Despite the various paces and capacities to walk in the group of day care attendees, most of them appreciated the  opportunity to get outdoors and walk. They had been accustomed to physical exercise previously, and the possibility to maintain this activity at the day care was highly appreciated. |
| Suwa, S., 2018, Japan | - Aki felt isolated since her oldest son and daughter‐in‐law (in their 70s) lived some distance away.  - In this year, her younger brother, the last of her remaining siblings, passed away. Aki wrote of growing loneliness and a feeling of solitude, “I'm lonely. Help me.”  - Aki began to refuse care from her family members and home care staff…physician and CM discussed the situation and were concerned that these new symptoms might be a side effect of Donepezil Hydrochloride. The medication was stopped, and the symptoms disappeared.  - While she felt the difficulties of living alone, she also wrote about her desire to live independently.  - “I can't show others my foolishness” and “I'm ignored” express that she was trying to be strong‐willed in front of others and was aware of being seen by others.  - Her grandchild's wedding was planned, but she thought, “I'm old and absentminded, so I probably won't be invited to the ceremony.” She felt, “I'm being left out.  - They come every day. They come in without permission. It's distressing.” “Even though I haven't asked them, they come without asking. I've got to stop it right away. I'll ask Chika about stopping them. |
| Svanström, R., 2015, Sweden | - The person seems to be familiar with the home and recognizes belongings. That which is outside the home appears to become unfamiliar and uncertain, which results in the person with dementia being reluctant to leave home alone. - The person with dementia with a manifest care need stops taking care of their home and themselves, and they seem to lose contact with people who are not close to them. Things that were once important in life, such as hobbies, clubs and travel, appear to lose meaning.  - Life is given meaning through social intercourse with others, which can explain the strong yearning for other people that the person with dementia with a manifest care need seems to have. It appears not to matter whether the visitor is a friend, a sibling, a child or just another fellow human being, who sits down, talks and listens.  - The participants express a wish for a longer visit from the caregivers for companionship and conversation.  - It becomes difficult for the person to recall their loved ones, and it seems to become even more difficult if there is no acquaintance to think about or meet. |
| Tranvåg, O., 2015, Norway | -...situations where HCPs made participants feel overlooked or devalued... - ...there are many times one is not allowed to do so . . . when those you meet don’t acknowledge you as an equal . . . making you feel small and unimportant ...  - ...when they are superficial . . . I can feel the difference . . . sensing that what you have to say is of real interest,..  - …be concerned about what you have to say …be treated as a real person . . . and allowed to be the center of  attention (of the HCPs) . . . and encouraged to express your concerns . . . and be listened to . . . |
| van der Roest, H. G., 2009, Netherlands | The highest proportions of unmet needs reported by persons with dementia concerned support for memory problems (10.2%), information about dementia, available care and treatment (9.9%), company (5.4%), and psychological distress (4.9%). |
| Van’t Leven, N., 2019, Netherlands | - In some cases, people tended to name specific activities during which they experienced a lack of self- sufficiency: hobbies, household activities, getting dressed, or operating devices in the house.  -I like watching television. I tend to turn it on for a bit in the morning, and then at the end of the afternoon. Well, that didn’t go well any more. |
| Wilkins, J. M., 2022, USA | - “Social engagement” preferences (e.g., regular contact with family, meeting new people, volunteering) were rated as significantly more important than preferences for other domains of everyday living (“autonomous choice,” “personal growth,” and “keeping a routine).  -...prior work that proxy ratings completed by care partners tended to significantly underestimate the importance of “social engagement” preferences for people with cognitive impairment (Wilkins et al., 2020). |
| Willis, R., 2020, Pakistan | - However, such (kind and positive attitude) was not always the case within families. Some family members became angry because they thought that the person with dementia was pretending.  - Overall, there was greater negativity from family members than from neighbors or community members. This could reflect carer stress, or possibly people with dementia interacting less frequently with community members than with family.  - Sometimes I don’t have any idea that it’s morning. To offer prayer is also very difficult.  - I usually ask a family member. . . . “Have I offered Zuhr [second daily] prayer?” If they see me while offering they say “Yes, you have done it”.  - Like in the Quran I did hifz [memorizing]. There are very long surat [verses] in the Quran so before that [the dementia] I have remembered all the surats but now I just forget. |
